# Chronic airflow obstruction and ambient particulate air pollution

**DOI:** 10.1136/thoraxjnl-2020-216223

**Published:** 2021-05-11

**Authors:** Andre F S Amaral, Peter G J Burney, Jaymini Patel, Cosetta Minelli, Filip Mejza, David M Mannino, Terence A R Seemungal, Padukudru Anand Mahesh, Li Cher Lo, Christer Janson, Sanjay Juvekar, Meriam Denguezli, Imed Harrabi, Emiel F M Wouters, Hamid Cherkaski, Kevin Mortimer, Rain Jogi, Eric D Bateman, Elaine Fuertes, Mohammed Al Ghobain, Wan Tan, Daniel O Obaseki, Asma El Sony, Michael Studnicka, Althea Aquart-Stewart, Parvaiz Koul, Herve Lawin, Asaad Ahmed Nafees, Olayemi Awopeju, Gregory E Erhabor, Thorarinn Gislason, Tobias Welte, Amund Gulsvik, Rune Nielsen, Louisa Gnatiuc, Ali Kocabas, Guy B Marks, Talant Sooronbaev, Bertrand Hugo Mbatchou Ngahane, Cristina Barbara, A Sonia Buist, The BOLD (Burden of Obstructive Lung Disease) Collaborative Research Group members, Hasan Hafizi

**Affiliations:** 1 National Heart and Lung Institute, Imperial College London, London, UK; 2 Centre for Evidence Based Medicine, 2nd Department of Internal Medicine, Jagiellonian University Medical College, Krakow, Poland; 3 Preventive Medicine and Environmental Health, University of Kentucky, Lexington, Kentucky, USA; 4 Clinical Medical Sciences, The University of the West Indies at St Augustine, Saint Augustine, Tunapuna-Piarco, Trinidad and Tobago; 5 Respiratory Medicine, JSS Medical College and Hospital, Mysore, Karnataka, India; 6 Department of Medicine, RCSI & UCD Malaysia Campus, Georgetown, Pulau Pinang, Malaysia; 7 Respiratory, Allergy and Sleep Research, Department of Medical Sciences, Uppsala University, Uppsala, Sweden; 8 Vadu Rural Health Program, King Edward Memorial Hospital Pune, Pune, Maharashtra, India; 9 Laboratoire de Physiologie et des Explorations Fonctionnelles, Universite de Sousse Faculte de Medecine de Sousse, Sousse, Tunisia; 10 Department of Respiratory Medicine, Maastricht University, Maastricht, The Netherlands; 11 Service de Epidemiologie et Medecine Preventive, Universite Badji Mokhtar Annaba Faculte de Medecine, Annaba, Algeria; 12 Clinical Sciences, Liverpool School of Tropical Medicine, Liverpool, UK; 13 Respiratory Medicine, Aintree University Hospitals NHS Foundation Trust, Liverpool, UK; 14 Lung Clinic, Tartu University Hospital, Tartu, Estonia; 15 Division of Respiratory Medicine, University of Cape Town, Rondebosch, Western Cape, South Africa; 16 Department of Medicine, King Saud bin Abdulaziz University for Health Sciences & King Abdullah International Medical Research Centre, Riyadh, Saudi Arabia; 17 iCAPTURE Centre, The University of British Columbia, Vancouver, Ontario, Canada; 18 Medicine, Obafemi Awolowo University, Ile-Ife, Osun, Nigeria; 19 Director, Epi-Lab, Khartoum, Sudan; 20 Department of Pulmonary Medicine, Paracelsus Medical University Salzburg, Salzburg, Austria; 21 Department of Internal Medicine, The University of the West Indies at Mona, Mona, Saint Andrew, Jamaica; 22 Pulmonary Medicine, SKIMS, Srinagar, Jammu and Kashmir, India; 23 Occupational and Environmental Health, University of Abomey-Calavi, Cotonou, Littoral, Benin; 24 Community Health Sciences, Aga Khan University, Karachi, Pakistan; 25 Department of Sleep, Landspitali University Hospital, Reykjavik, UK; 26 Medicine, University of Iceland, Reykjavik, Iceland; 27 Respiratory Medicine, Medizinische Hochschule Hannover, Hannover, Germany; 28 Department of Thoracic Medicine, Haukeland University Hospital, Bergen, Norway; 29 Department of Clinical Science, University of Bergen, Bergen, Norway; 30 Nuffield Department of Population Health, Oxford University, Oxford, UK; 31 Department of Chest Disease, Cukurova University, School of Medicine, Adana, Turkey; 32 Respiratory and Environmental Epidemiology, Woolcock Institute of Medical Research, Glebe, New South Wales, Australia; 33 South Western Sydney Clinical School, University of New South Wales, Sydney, New South Wales, Australia; 34 Department of Respiratory Medicine, National Center for Cardiology and Internal Medicine, Bishkek, Kyrgyzstan; 35 Internal Medicine, Douala General Hospital, Douala, Cameroon; 36 Institute of Environmental Health, Lisbon Medical School, Lisbon University, Lisboa, Portugal; 37 Pulmonary and Critical Care Medicine, Oregon Health & Science University, Portland, Oregon, USA

**Keywords:** COPD epidemiology

## Abstract

Smoking is the most well-established cause of chronic airflow obstruction (CAO) but particulate air pollution and poverty have also been implicated. We regressed sex-specific prevalence of CAO from 41 Burden of Obstructive Lung Disease study sites against smoking prevalence from the same study, the gross national income per capita and the local annual mean level of ambient particulate matter (PM_2.5_) using negative binomial regression. The prevalence of CAO was not independently associated with PM_2.5_ but was strongly associated with smoking and was also associated with poverty. Strengthening tobacco control and improved understanding of the link between CAO and poverty should be prioritised.

## Introduction

The most important cause of chronic airflow obstruction (CAO) is tobacco smoking. The Global Burden of Disease programme has suggested that air pollution is second only to smoking in determining loss of disability-adjusted life-years due to chronic respiratory disease.^1^ Evidence for this was obtained by applying the risk of disease associated with air pollution exposure, as estimated from various studies, to the known distribution of fine particulate matter (PM_2.5_) across the world.

In this analysis, we investigated the ecological association (ie, using aggregated data)^2^ between the prevalence of CAO, as estimated from a large multisite study, and levels of ambient PM_2.5_.

## Methods

The prevalence of CAO and the prevalence of smoking were estimated for 41 sites of the Burden of Obstructive Lung Disease (BOLD) study (online supplemental file for details).^3^ The level of poverty of each site was estimated from the gross national income (GNI) per capita at the time of the survey, using data from the World Bank.^4^ Annual mean PM_2.5_ levels (all composition, and dust and sea-salt removed (DSSR)) for each site coordinates and a 10 km radius buffer (site as centre) were obtained from a public dataset.^5 6^

10.1136/thoraxjnl-2020-216223.supp1 Supplementary data

The unit of our analysis was the site, and the analysis was stratified by sex (online supplemental file for details).

## Results

The prevalence of CAO across sites ranged from 3.5% to 23.2% in men, and from 2% to 19.4% in women (table 1). As expected, the prevalence of CAO was substantially lower among never smokers (online supplemental table S1).

**Table 1:** Survey date, prevalence of chronic airflow obstruction (CAO) and smoking in men and women, gross national income (GNI) per capita and annual mean PM_2.5_ levels for the 41 sites of the Burden of Obstructive Lung Disease study

Site	Mid-date of survey	CAO in men (%)	CAO in women (%)	Ever smoking prevalence in men (%)	Ever smoking prevalence in women (%)	GNI per capita, PPP (current international $)	PM_2.5_ (all composition) (μg/m^3^)	PM_2.5_ (all composition) 10 km radius buffer (μg/m^3^)	PM_2.5_ (dust and sea-salt removed) (μg/m^3^)	PM_2.5_ (dust and sea-salt removed) 10 km radius buffer (μg/m^3^)
Albania (Tirana)	17/02/2013	12.8	4.2	63.0	11.4	10 750	25	16.7	15	10.0
Algeria (Annaba)	28/06/2012	9.3	4.5	76.5	0.7	13 230	21	14.5	8	5.5
Australia (Sydney)	30/07/2006	7.9	13.8	60.8	47.5	32 970	7	6.6	4	4.0
Austria (Salzburg)	11/01/2005	12.8	19.4	64.4	44.3	34 940	23	18.1	20	16.0
Benin (Sèmè-Kpodji)	06/03/2014	6.6	8.1	4.6	0	2100	28	24.8	13	11.7
Cameroon (Limbe)	11/02/2015	6.3	4.3	35.9	2.9	3390	41	37.0	20	18.0
Canada (Vancouver)	30/12/2003	12.8	12.0	66.0	50.3	31 540	5	5.9	4	5.2
China (Guangzhou)	26/11/2002	9.9	6.3	81.4	6.3	3520	40	38.7	39	37.2
England (London)	27/02/2007	16.1	15.8	71.8	57.1	35 240	15	15.3	13	13.2
Estonia (Tartu)	25/02/2009	8.7	5.2	63.8	31.5	19 880	12	10.7	11	9.4
Germany (Hannover)	16/07/2005	10.0	7.8	73.1	50	32 350	20	20.0	18	18.2
Iceland (Reykjavik)	28/04/2005	8.9	13.3	70.7	61.3	35 470	4	4.1	1	1.4
India (Kashmir)	11/03/2011	17.3	15.4	76.4	28.8	4580	33	33.6	26	26.7
India (Mumbai)	13/05/2007	6.2	7.9	15.6	0	3610	39	40.3	34	34.6
India (Mysore)	08/04/2012	11.2	5.5	22.1	1.4	4850	22	22.1	19	19.9
India (Pune)	24/09/2009	5.8	6.7	20.9	0.3	4000	45	44.9	40	39.3
Jamaica	01/03/2015	10.3	7.5	64.2	18.5	8280	8	6.5	3	2.3
Kyrgyzstan (Chui)	04/07/2013	13.9	7.9	77.9	7.5	3050	19	18.5	9	8.9
Kyrgyzstan (Naryn)	02/07/2013	11.0	4.7	60.4	2.4	3050	24	23.5	7	7.0
Malawi (Blantyre)	24/10/2013	6.9	9.1	30.6	2.5	1120	11	11.1	11	10.5
Malawi (Chikwawa)	15/04/2015	18.0	9.4	48.6	11.3	1190	16	15.5	15	14.5
Malaysia (Penang)	15/08/2013	4.4	3.4	49.7	0	23 470	33	22.8	30	20.8
Morocco (Fes)	17/10/2010	11.9	7.5	59.3	1.0	6240	24	19.1	6	5.0
Netherlands (Maastricht)	30/06/2008	19.0	17.2	73.7	60.3	45 110	14	14.1	13	12.6
Nigeria (Ile-Ife)	10/09/2011	7.5	6.7	23.4	3.7	4920	30	34.3	15	17.1
Norway (Bergen)	13/08/2005	14.8	10.2	71.0	57.8	48 300	7	6.7	4	4.4
Pakistan (Karachi)	18/01/2015	14.6	6.5	48.6	8.0	5050	68	67.9	17	17.0
Philippines (Manila)	25/12/2005	13.0	5.2	83.9	31.1	5050	28	27.6	21	20.6
Philippines (Nampicuan-Talugtug)	21/08/2007	16.3	12.3	77.0	30.1	5710	13	12.6	10	10.2
Poland (Krakow)	10/05/2005	15.0	12.3	79.4	43.8	13 650	37	35.8	34	33.5
Portugal (Lisbon)	26/08/2008	13.9	9.5	61.6	22.1	25 590	14	10.9	8	6.5
Saudi Arabia (Riyadh)	06/10/2012	3.5	2.8	48.3	2.2	51 250	64	64.1	13	13.0
South Africa (Uitsig-Ravensmead)	05/04/2005	23.8	16.2	84.4	57.9	9610	8	7.5	5	4.5
Sri Lanka	28/09/2013	11.7	3.9	48.9	0.2	10 370	15	14.2	10	9.3
Sudan (Gezeira)	25/04/2016	5.6	6.0	47.8	1.4	4260	40	40.2	5	5.0
Sudan (Khartoum)	25/03/2013	10.4	10.0	38.4	2.9	2690	39	38.4	6	5.7
Sweden (Uppsala)	20/03/2007	10.2	8.3	68.5	52.7	41 850	8	6.7	7	5.7
Trinidad & Tobago	23/06/2015	6.6	6.7	51.3	12.0	33 280	7	7.1	1	1.0
Tunisia (Sousse)	01/11/2010	8.4	2.0	79.9	9.1	9750	20	17.3	6	5.3
Turkey (Adana	30/12/2003	19.8	9.1	81.0	30.5	9430	32	27.7	17	14.8
USA (Lexington, KY)	13/02/2006	13.6	16.2	78.6	54.3	47 160	11	9.9	10	9.7

PM_2.5_, particulate matter <2.5 µm aerodynamic diameter; PPP, Purchasing power parity.

The prevalence of smoking varied from 4.6% to 84.4% in men and from 0% to 61.3% in women. The levels of all composition PM_2.5_ ranged from 4 µg/m^3^ in Reykjavik (Iceland) to 68 µg/m^3^ in Karachi (Pakistan). The GNI varied from $1120 in Malawi to $51 250 in Saudi Arabia (table 1).

**Figure 1:**
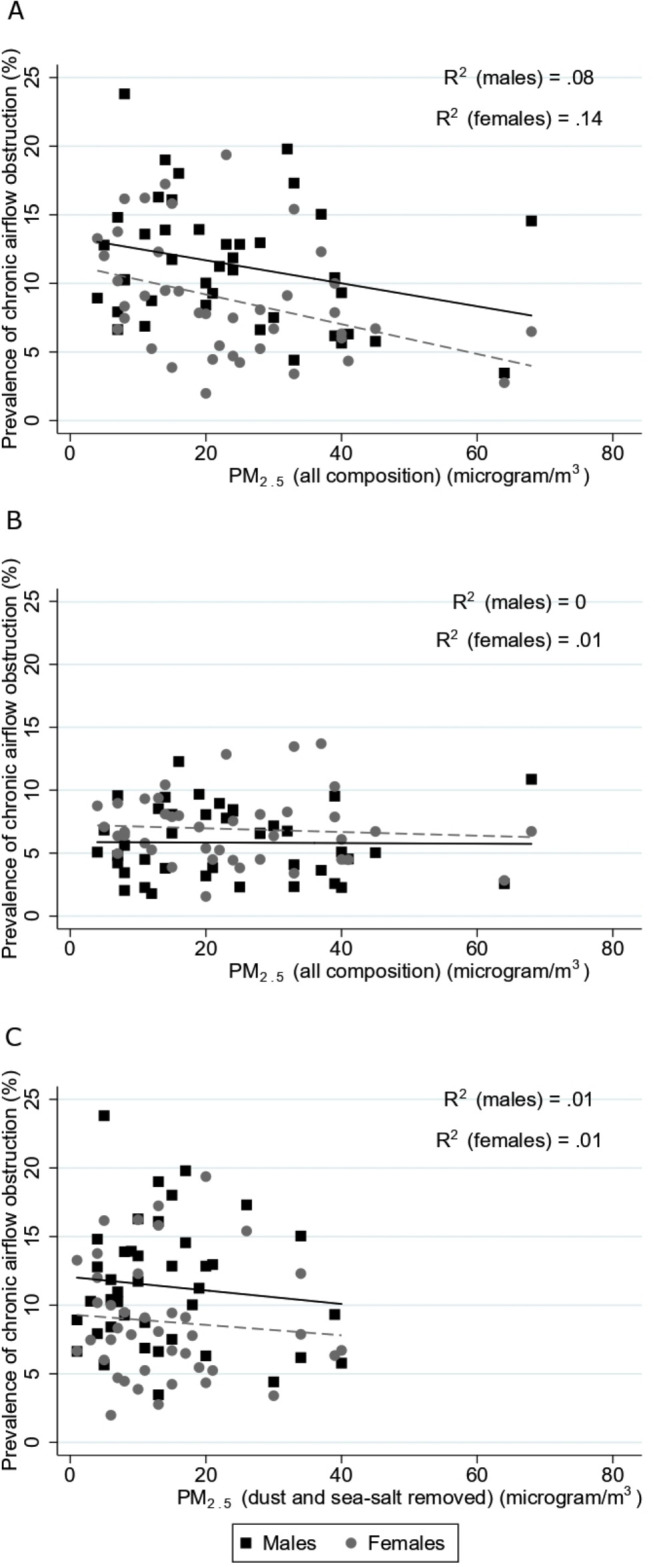
Relation between prevalence of chronic airflow obstruction and annual mean levels of (a) PM_2.5_ (all composition, μg/m^3^) for the whole sample, (B) PM_2.5_ (all composition, μg/m^3^) for never smokers and (C) PM_2.5_ (dust and sea-salt removed, μg/m^3^) for the whole sample.

Lower PM_2.5_ levels were weakly correlated with a higher prevalence of CAO, in both sexes (figure 1A). Among never smokers (figure 1B) and when using DSSR PM_2.5_, there was no correlation (figure 1C).

In both sexes, the prevalence of CAO was strongly positively associated with smoking and negatively associated with GNI. There was no association of prevalence of CAO with levels of PM_2.5_ (all composition) (table 2). The sensitivity analyses using all composition PM_2.5_ for a 10 km radius buffer and using DSSR PM_2.5_ showed no substantive difference from the main analysis (online supplemental tables S2–S4).

**Table 2:** Ecological negative binomial regression of chronic airflow obstruction against log(GNI), smoking and log(PM_2.5_), by sex

Variable	men			women		
	Rate ratio	95% CI	P value	Rate ratio	95% CI	P value
Smoking	4.17	2.40 to 7.26	<0.001	11.3	5.64 to 22.6	<0.001
Log(GNI)	0.90	0.81 to 0.99	0.04	0.83	0.73 to 0.94	0.003
Log(PM_2.5_)	0.92	0.78 to 1.07	0.28	1.05	0.89 to 1.25	0.55

GNI, gross national income; PM2.5, particulate matter <2.5µm aerodynamic diameter.

## Discussion

We were unable to show evidence of an ecological association between the prevalence of CAO and annual mean levels of PM_2.5_, although we have shown clear independent associations with the prevalence of smoking and GNI.

Our findings suggest that PM_2.5_ is unlikely to have a substantial effect on the prevalence of CAO. We have previously shown that indoor burning of solid fuels, another source of PM_2.5_, is also unlikely to be substantially associated with CAO,^7^ a conclusion supported by the findings of three large Chinese studies.^8–10^ Our findings are compatible with the large European ESCAPE project, which showed little evidence of an effect of any pollutant on the FEV_1_/FVC or its change over time.^11^

This analysis has several strengths. The aggregate data on prevalence of CAO and smoking were taken directly from the BOLD study. Spirometry was post-bronchodilator, and its quality was assured with a strong training programme and regular review of all spirograms in a quality control centre.

All ecological analyses have potential weaknesses. One is the temptation to ascribe the associations observed at the site level to similar associations at an individual level. In this instance, there is independent analysis showing the association of CAO with smoking^12^ and poverty^13^ at the individual level within the BOLD study.

Ecological analyses are also prone to confounding. There are strong ecological associations between the prevalence of smoking, GNI and PM_2.5_. The poorer countries have fewer smokers, less CAO and greater pollution levels. This probably explains the negative association of CAO with PM_2.5_ in the population as a whole, which was not seen for never smokers (figure 1B), or with DSSR PM_2.5_, or in the regression analysis adjusted for smoking prevalence and GNI.

Ecological analyses can be misleading if the average exposure in a site does not represent the exposure of those with the disease.^14^ Although there may be differences in pollution exposure within each site, these are likely to be small compared with the larger variation between sites, which ranged from 4 µg/m^3^ in Reykjavik (Iceland) to 68 µg/m^3^ in Karachi (Pakistan). It is unlikely that anyone living in Karachi will have exposure to ambient PM_2.5_ lower than any of those living in Reykjavik. The wide variation in income across sites is probably less well represented by GNI. Using the same estimate of GNI for rural and urban areas is likely to lead to more substantial errors than the approximations made for PM_2.5_. Nevertheless, we have found an association between poverty and CAO both at the ecological and individual levels in the BOLD study,^13^ and it is likely that the imprecision introduced here by using GNI to represent the site income has reduced the strength of association with CAO.

These results do not imply that air pollution is not harmful to lung growth in utero and during childhood, lung health or general health, and we clearly do not address in this study the potential of PM_2.5_ to cause other pathologies or to trigger acute exacerbations of disease. We cannot exclude the possibility that the toxicology of PM_2.5_ varies geographically, that a component of PM_2.5_ causes CAO but it is not always present, or that there is another pollutant that is highly correlated with PM_2.5_ in some sites that causes CAO. Several researchers have suggested that the properties^15^ or sources^16^ of particles may also be important in determining their effects.

This ecological study shows that, after adjustment for smoking and GNI, ambient PM_2.5_ is unlikely to explain a substantial amount of the prevalence of CAO, while the ecological association of smoking with CAO is strong and the association of poverty with CAO indicates that this is also likely to play an important role in its origins.
