# Supplementary material for: Chronic airflow obstruction and ambient particulate air pollution
Source: Thorax. 2021 May 11;76(12):1236–41. doi: 10.1136/thoraxjnl-2020-216223 (PMC8606424; doi:10.1136/thoraxjnl-2020-216223)
Supplement: Supplementary data [file thoraxjnl-2020-216223supp001.pdf]

Online supplement

Chronic airflow obstruction and ambient particulate air pollution

André F.S. Amaral[1*], Peter Burney[1*], Jaymini Patel[1], Cosetta Minelli[1], Filip Mejza[2], David Mannino[3], Terence Seemungal[4], Mahesh PA[5], Li Cher Loh[6], Christer Janson[7], Sanjay Juvekar[8], Meriam Denguezli[9], Imed Harrabi[10], Emiel F.M. Wouters[11], Hamid Chersaki[12], Kevin Mortimer[13], Rain Jögi[14], Eric Bateman[15], Elaine Fuertes[1], Mohamed Al Ghobain[16], Wan Tan[17], Daniel Obaseki[18], Asma El Sony[19], Michael Studnicka[20], Althea Aquart-Stewart[21], Parvaiz Koul[22], Hervé Lawin[23], Asaad Ahmed Nafees[24], Olayemi Awopeju[18], Gregory Erhabor[18], Thorarinn Gislason[25,26], Tobias Welte[27], Amund Gulsvik[28], Rune Nielsen[28,29], Louisa Gnatiuc[30], Ali Koçabas[31], Guy Marks[32,33], Talant Sooronbaev[34], Bertrand Hugo Mbatchou Ngahane[35], Cristina Bárbara[36], A Sonia Buist[37]

*Co-first authors

1 National Heart and Lung Institute, Imperial College London, London, UK

2 Centre for Evidence Based Medicine, 2nd Department of Internal Medicine, Jagiellonian University Medical College, Kraków, Poland

3 University of Kentucky, Lexington, KY, USA

4 Faculty of Medical Sciences, University of West Indies, St Augustine, Trinidad and Tobago

5 Department of Respiratory Medicine, JSS Medical College, Mysore, India

6 Department of Medicine, RCSI & UCD Malaysia Campus, Penang, Malaysia

7 Department of Medical Sciences: Respiratory, Allergy and Sleep Research, Uppsala University, Uppsala, Sweden

8 Vadu Rural Health Program (VRHP), KEM Hospital Research Centre, Pune, India

9 Laboratoire de Physiologie et des Explorations Fonctionnelles, Faculté de Médecine de Sousse, Université de Sousse, Sousse, Tunisia

10 Faculté de Médecine, Université de Sousse, Sousse, Tunisia

11 Maastricht University Medical Center, Maastricht, The Netherlands

12 Faculté de Médecine de Annaba, Service de Epidémiologie et Médecine Préventive, El Hadjar, Algeria

13 Liverpool School of Tropical Medicine, Liverpool, UK

14 Lung Clinic, Tartu University Hospital, Tartu, Estonia

15 University of Cape Town Lung Institute, Cape Town, South Africa

16 Department of Medicine, King Saud bin Abdulaziz University for Health Sciences & King Abdullah International Medical Research Centre, Riyadh, Saudi Arabia

17 Centre for Heart Lung Innovation, University of British Columbia, Vancouver, BC, Canada

18 Obafemi Awolowo University, Ile-Ife, Nigeria

19 The Epidemiological Laboratory, Khartoum, Sudan

20 Department of Pneumology, Paracelsus Medical University, Salzburg, Austria

21 University of the West Indies, Kingston, Jamaica

22 Sher-i-Kashmir Institute of Medical Sciences, Srinagar, J&K, India

23 Occupational and Environmental Health, University of Abomey Calavi, Cotonou, Benin

24 Department of Community Health Sciences, Aga Khan University, Karachi, Pakistan

25 Department of Sleep, Landspítali - The National University Hospital of Iceland, Reykjavik, Iceland

26 University of Iceland, Faculty of Medicine, Reykjavik, Iceland

27 Hannover Medical School, Hannover, Germany

28 Department of Thoracic Medicine, Haukeland University Hospital, Bergen, Norway

29 Department of Clinical Science, University of Bergen, Bergen, Norway

30 Nuffield Department of Population Health, Oxford University, Oxford, UK

31 Department of Chest Disease. Cukurova University School of Medicine. Adana, Turkey

32 Woolcock Institute of Medical Research, Sydney, NSW, Australia

33 South Western Sydney Clinical School, University of New South Wales, Sydney, Australia

34 Department of Respiratory Medicine, National Center for Cardiology and Internal Medicine, Bishkek, Kyrgyzstan

35 Douala General Hospital, Douala, Cameroon

36 Institute of Environmental Health, Lisbon Medical School, Lisbon University, Lisbon, Portugal

37 Oregon Health & Science University, Portland, OR, USA

Corresponding author:

Andre F.S. Amaral

National Heart and Lung Institute, Imperial College London

London SW3 6LR, UK

a.amaral@imperial.ac.uk

Table of content

BOLD study details ---------- page 4

$PM_{2.5}$ concentrations ---------- page 4

Statistical analysis ---------- page 5

References ---------- page 5

Table S1 ---------- page 7

Table S2 ---------- page 8

Table S3 ---------- page 8

Table S4 ---------- page 8

Amaral AFS, *et al. Thorax* 2021; 76:1236–1241. doi: 10.1136/thoraxjnl-2020-216223

BOLD study details

The BOLD study has been described in detail elsewhere[1]. Briefly, representative samples of the general population over the age of 40 were drawn from a sample of sites taken from around the world. These were drawn so as to represent most of the regions defined by the Global Burden of Disease (the exceptions were Latin America which had a separate study, the high-income countries of Asia, and Oceania) with a bias towards larger regions such as South Asia. All sites received approval from their local ethics committee, and participants provided informed consent. Spirometry was conducted before and after a bronchodilator (200 μg Salbutamol inhaled via a spacer) using a forced manoeuvre and an EasyOne spirometer (ndd Medizintechnik AG, Zurich, Switzerland). All spirometry data were reviewed for quality control during the study in either Salt Lake City or London. CAO was defined as a post-bronchodilator ratio of the forced expiratory volume in the first second (FEV_1) to the forced vital capacity (FVC), less than the lower limit of normal, which was determined using the equations for white Americans in the NHANES III study[2]. Information from participants on several risk factors, including whether they had ever smoked, was collected using a standardised questionnaire.

$PM_{2.5}$ concentrations

The concentrations of annual mean $PM_{2.5}$ were downloaded from a freely available resource on the Atmospheric Composition Analysis Group website.[3] Briefly, global ground-level $PM_{2.5}$ concentrations were estimated by combining aerosol optical depth data from satellites with simulation-based data from a chemical transport model. These were then calibrated to ground-based observations of $PM_{2.5}$ using a geographically weighted regression, which allows the estimation of local levels.[4] We used data at a spatial resolution of 0.01° latitude by 0.01° longitude.

Statistical analysis

The unit of our ecological analysis was the site, and the analysis was performed separately for males and females. The counts of the male and female cases of CAO in each site were regressed separately in negative binomial models, with the local sample size as an offset. The analyses included the prevalence of male, or female, smoking in the site as measured from the BOLD sample, the GNI and the level of $PM_{2.5}$ (all composition) for the site. Both GNI and $PM_{2.5}$ were log transformed as there is evidence in each case of a non-linear relationship with chronic lung disease[5,6]. We conducted a series of sensitivity analyses in which we re-run the main analysis: 1) using $PM_{2.5}$ (all composition) estimates for a 10-km radius buffer; and 2) using dust and sea-salt removed $PM_{2.5}$ instead of all composition $PM_{2.5}$.

References

1. Buist AS, Vollmer WM, Sullivan SD, et al. The Burden of Obstructive Lung Disease Initiative (BOLD): rationale and design. *Copd* 2005; **2**(2): 277-83.

2. Hankinson JL, Odencrantz JR, Fedan KB. Spirometric reference values from a sample of the general U.S. population. *American journal of respiratory and critical care medicine* 1999; **159**(1): 179-87.

3. Atmospheric Composition Analysis Group. Surface PM2.5. http://fizz.phys.dal.ca/~atmos/martin/?page_id=140 (accessed 18 February 2020).

4. van Donkelaar A, Martin RV, Brauer M, et al. Global Estimates of Fine Particulate Matter using a Combined Geophysical-Statistical Method with Information from Satellites, Models, and Monitors. *Environ Sci Technol* 2016; **50**(7): 3762-72.

5. Burney P, Jithoo A, Kato B, et al. Chronic obstructive pulmonary disease mortality and prevalence: the associations with smoking and poverty--a BOLD analysis. *Thorax* 2014; **69**(5): 465-73.

6. Burnett RT, Pope CA, Ezzati M, et al. An integrated risk function for estimating the global burden of disease attributable to ambient fine particulate matter exposure. *Environmental health perspectives* 2014; **122**(4): 397-403.

Table S1. Sample size and prevalence of chronic airflow obstruction (CAO) in the population and in never smokers by site and sex.

	Males			Females		
Site	N	CAO %	CAO % in never smokers	N	CAO %	CAO % in never smokers
Albania (Tirana)	467	12.8	2.3	472	4.2	3.8
Algeria (Annaba)	442	9.3	3.8	448	4.5	4.5
Australia (Sydney)	265	7.9	4.8	276	13.8	9.0
Austria (Salzburg)	685	12.8	7.8	573	19.4	12.9
Benin (Sèmè-Kpodji)	302	6.6	6.6	396	8.1	8.1
Cameroon (Limbe)	206	6.3	4.5	138	4.3	4.5
Canada (Vancouver)	344	12.8	6.8	483	12.0	7.1
China (Guangzhou)	236	9.9	2.3	237	6.3	4.5
England (London)	323	16.1	6.6	354	15.8	7.9
Estonia (Tartu)	309	8.7	1.8	305	5.2	5.3
Germany (Hannover)	349	10.0	3.2	334	7.8	5.4
Iceland (Reykjavik)	403	8.9	5.1	354	13.3	8.8
India (Kashmir)	416	17.3	4.1	344	15.4	13.5
India (Mumbai)	275	6.2	2.6	165	7.9	7.9
India (Mysore)	258	11.2	9.0	348	5.5	5.2
India (Pune)	502	5.8	5.0	343	6.7	6.7
Jamaica	243	10.3	3.4	335	7.5	6.6
Kyrgyzstan (Chui)	280	13.9	9.7	611	7.9	7.1
Kyrgyzstan (Naryn)	328	11.0	8.5	531	4.7	4.4
Malawi (Blantyre)	160	6.9	4.5	242	9.1	9.3
Malawi (Chikwawa)	222	18.0	12.3	212	9.4	8.0
Malaysia (Penang)	340	4.4	2.3	323	3.4	3.4
Morocco (Fes)	354	11.9	8.3	414	7.5	7.6
Netherlands (Maastricht)	300	19.0	3.8	290	17.2	10.4
Nigeria (Ile-Ife)	346	7.5	7.2	538	6.7	6.4
Norway (Bergen)	324	14.8	9.6	334	10.2	5.0
Pakistan (Karachi)	268	14.6	10.0	339	6.5	6.7
Philippines (Manila)	378	13.0	6.6	515	5.2	4.5
Philippines (Nampicuan-Talugtug)	356	16.3	8.5	366	12.3	9.4
Poland (Krakow)	266	15.0	3.6	260	12.3	13.7
Portugal (Lisbon)	331	13.9	9.4	380	9.5	8.1
Saudi Arabia (Riyadh)	375	3.5	2.6	325	2.8	2.8
South Africa (Uitsig-Ravensmead)	315	23.8	2.0	532	16.2	6.7
Sri Lanka	460	11.7	8.1	568	3.9	3.9
Sudan (Gezeira)	301	5.6	5. 1	283	6.0	6.1
Sudan (Khartoum)	307	10.4	9.5	210	10.0	10.3
Sweden (Uppsala)	283	10.2	5.6	264	8.3	6.4
Trinidad & Tobago	437	6.6	4.2	660	6.7	6.4
Tunisia (Sousse)	309	8.4	8.1	352	2.0	1.6
Turkey (Adana)	389	19.8	6.8	417	9.1	8.3
USA (Lexington, KY)	206	13.6	2.3	302	16.2	5.8

N, sample size.

Amaral AFS, *et al. Thorax* 2021; 76:1236–1241. doi: 10.1136/thoraxjnl-2020-216223

Table S2. Sensitivity analysis – as for table 3, with estimates of $PM_{2.5}$ for a 10-km radius buffer.

	Males			Females		
Variable	Rate ratio	95%CI	*P*	Rate ratio	95%CI	*P*
Smoking	4.17	2.39-7.29	< 0.001	11.1	5.66-21.8	< 0.001
Log(GNI)	0.90	0.81-0.99	0.048	0.83	0.73-0.94	0.003
Log($PM_{2.5}$)	0.93	0.79-1.09	0.37	1.05	0.89-1.25	0.55

GNI, gross national income per capita; $PM_{2.5}$, particulate matter <2.5μm aerodynamic diameter.

Table S3. Sensitivity analysis – as for table 3, with estimates of $PM_{2.5}$ with dust and sea-salt removed.

	Males			Females		
Variable	Rate ratio	95%CI	*P*	Rate ratio	95%CI	*P*
Smoking	4.38	2.51-7.62	< 0.001	10.4	5.52-19.6	< 0.001
Log(GNI)	0.92	0.83-1.02	0.1	0.83	0.73-0.94	0.003
Log($PM_{2.5}$)	1.04	0.92-1.18	0.48	1.03	0.92-1.16	0.59

GNI, gross national income per capita; $PM_{2.5}$, particulate matter <2.5μm aerodynamic diameter.

Table S4. Sensitivity analysis – as for table 3, with estimates of $PM_{2.5}$ with dust and sea-salt removed for a 10-km radius buffer.

	Males			Females		
Variable	Rate ratio	95%CI	*P*	Rate ratio	95%CI	*P*
Smoking	4.41	2.53-7.68	< 0.001	10.3	5.47-19.4	< 0.001
Log(GNI)	0.92	0.84-1.02	0.11	0.83	0.73-0.94	0.004
Log($PM_{2.5}$)	1.06	0.93-1.19	0.39	1.03	0.92-1.16	0.60

GNI, gross national income per capita; $PM_{2.5}$, particulate matter <2.5μm aerodynamic diameter.

Amaral AFS, *et al. Thorax* 2021; 76:1236–1241. doi: 10.1136/thoraxjnl-2020-216223
